# Supplementary material for: INFEKTA—An agent-based model for transmission of infectious diseases: The COVID-19 case in Bogotá, Colombia
Source: PLoS One. 2021 Feb 19;16(2):e0245787. doi: 10.1371/journal.pone.0245787 (PMC7894857; doi:10.1371/journal.pone.0245787)

Infectious disease states

S := Susceptible
E := Exposed
I_A := Asymptomatic-Infected
I_S := Seriously-Infected
I_S := Critically-Infected
R := Recovered
M := Immune
D := Dead

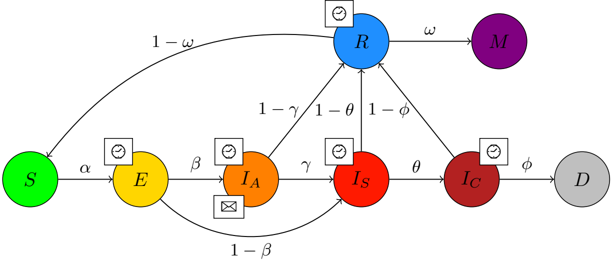

Supplement: S1 File — A repository containing the source code of the simulator and a technical report explaining the modeling methodology is available at INFEKTA github. (ZIP) [file pone.0245787.s001.zip › images/Fig1.pdf]
