# Supplementary material for: INFEKTA—An agent-based model for transmission of infectious diseases: The COVID-19 case in Bogotá, Colombia
Source: PLoS One. 2021 Feb 19;16(2):e0245787. doi: 10.1371/journal.pone.0245787 (PMC7894857; doi:10.1371/journal.pone.0245787)

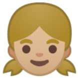

Age: Child
Gender: Female
IP: School
UPZ: 1

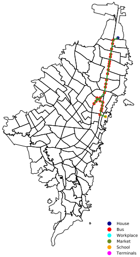

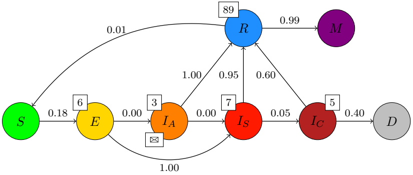

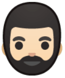

Age: Adult
Gender: Male
IP: Workplace
UPZ: 47

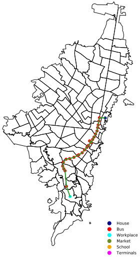

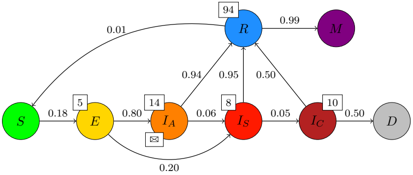

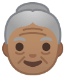

Age: Older
Gender: Female
IP: Workplace
UPZ: 90

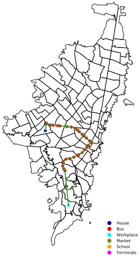

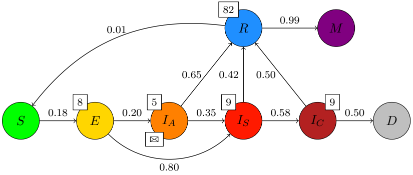

Supplement: S1 File — A repository containing the source code of the simulator and a technical report explaining the modeling methodology is available at INFEKTA github. (ZIP) [file pone.0245787.s001.zip › images/Fig3.pdf]
