# Supplementary figures and images for: INFEKTA—An agent-based model for transmission of infectious diseases: The COVID-19 case in Bogotá, Colombia
Source: PLoS One. 2021 Feb 19;16(2):e0245787. doi: 10.1371/journal.pone.0245787 (PMC7894857; doi:10.1371/journal.pone.0245787)

● House ● Bus ● Workplace ● Market ● School ● Terminals

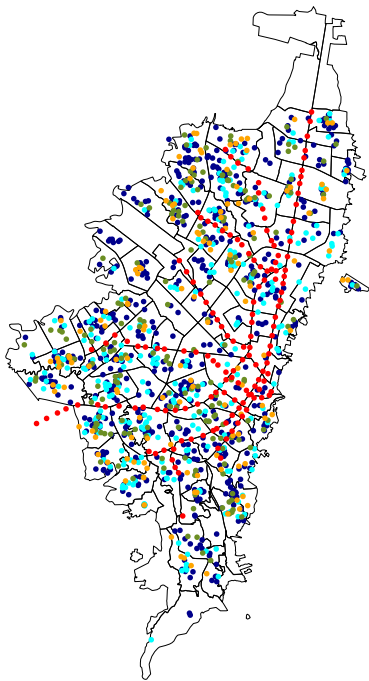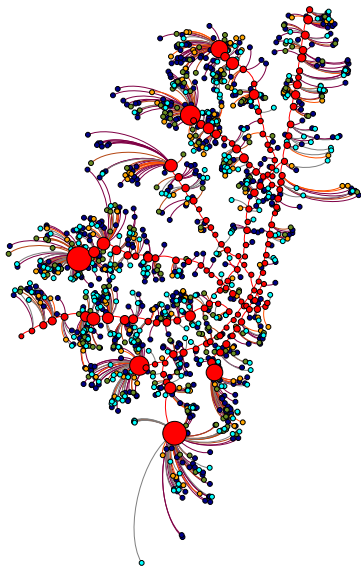

Supplement: S1 File — A repository containing the source code of the simulator and a technical report explaining the modeling methodology is available at INFEKTA github. (ZIP) [file pone.0245787.s001.zip › images/Fig2.pdf]

Individuals

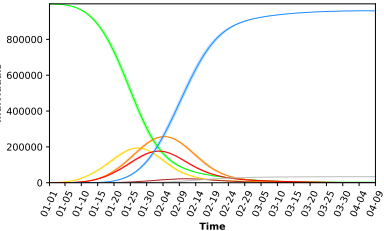

Tick: 02-03

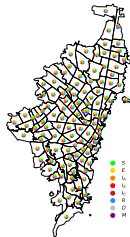

Tick: 03-07

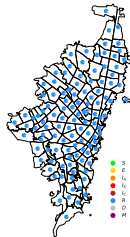

Supplement: S1 File — A repository containing the source code of the simulator and a technical report explaining the modeling methodology is available at INFEKTA github. (ZIP) [file pone.0245787.s001.zip › images/Fig4.pdf]

Tick: 02-03

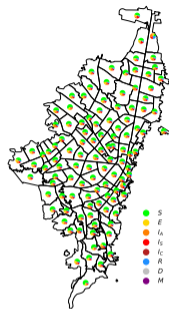

Tick: 02-03

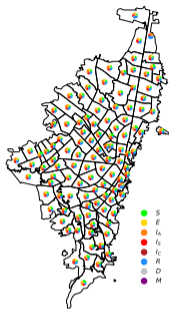

Tick: 02-03

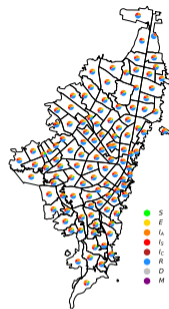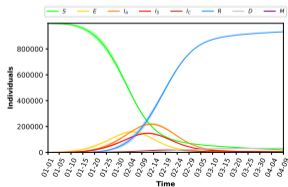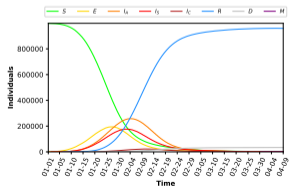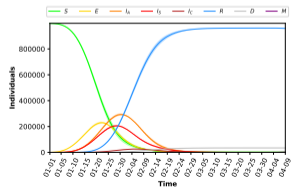

Supplement: S1 File — A repository containing the source code of the simulator and a technical report explaining the modeling methodology is available at INFEKTA github. (ZIP) [file pone.0245787.s001.zip › images/Fig5.pdf]

Tick: 02-03

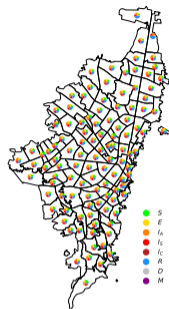

Tick: 02-03

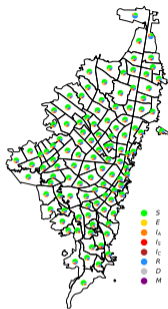

Tick: 02-03

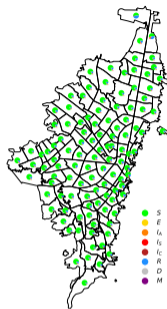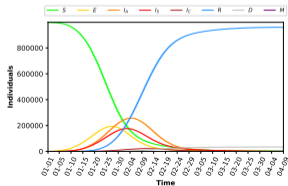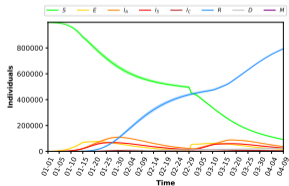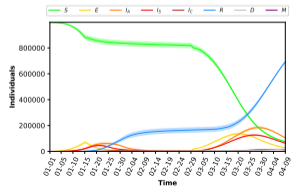

Supplement: S1 File — A repository containing the source code of the simulator and a technical report explaining the modeling methodology is available at INFEKTA github. (ZIP) [file pone.0245787.s001.zip › images/Fig6.pdf]

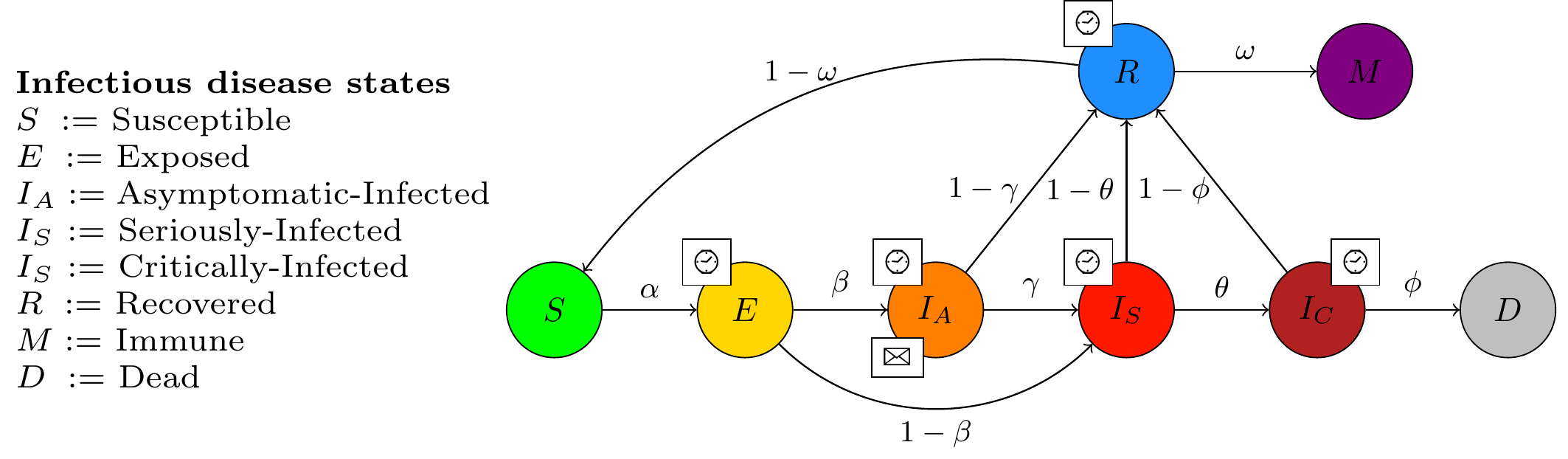

Supplement: S1 File — A repository containing the source code of the simulator and a technical report explaining the modeling methodology is available at INFEKTA github. (ZIP) [file pone.0245787.s001.zip › images/Fig1.tiff]

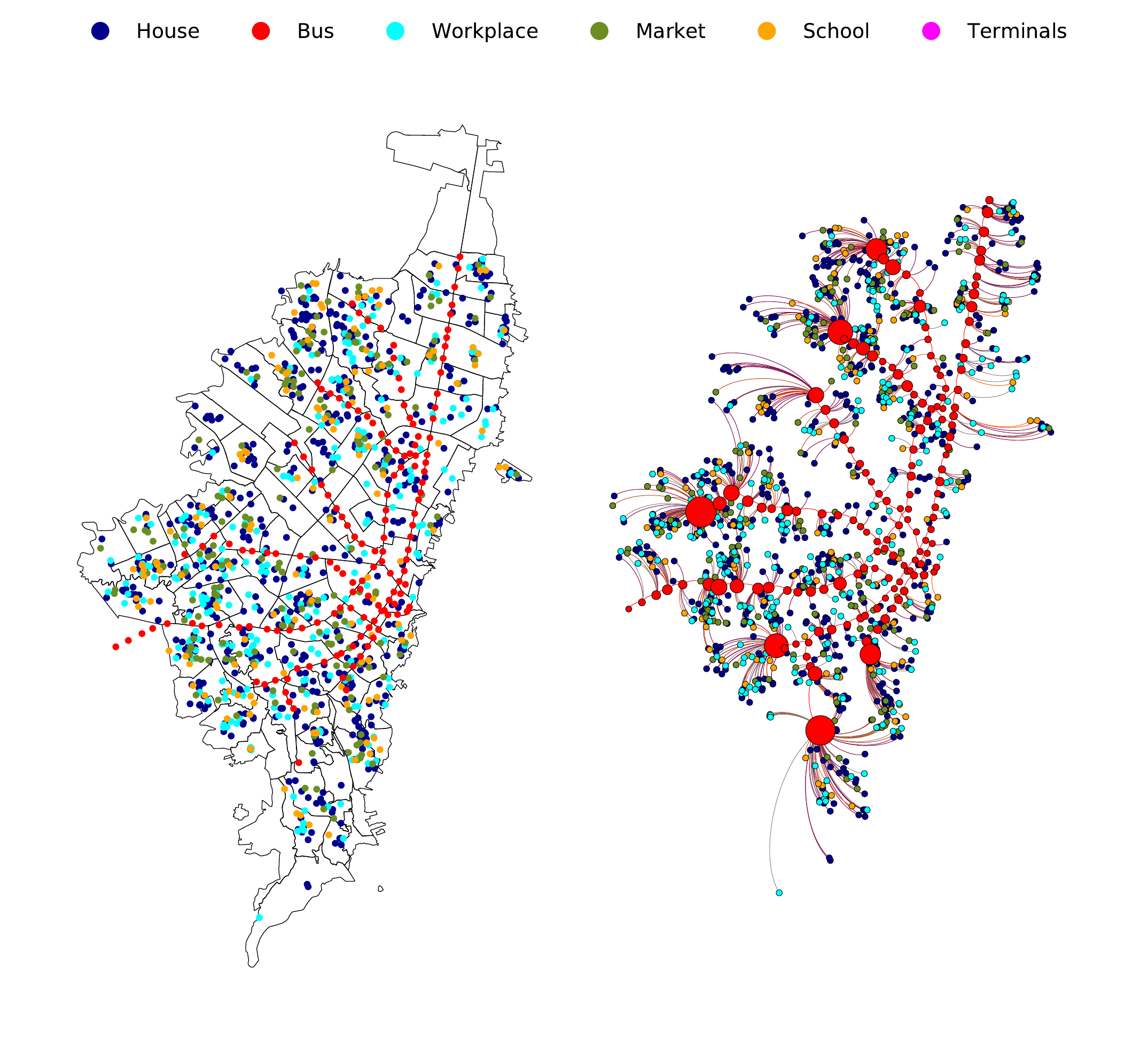

Supplement: S1 File — A repository containing the source code of the simulator and a technical report explaining the modeling methodology is available at INFEKTA github. (ZIP) [file pone.0245787.s001.zip › images/Fig2.tiff]

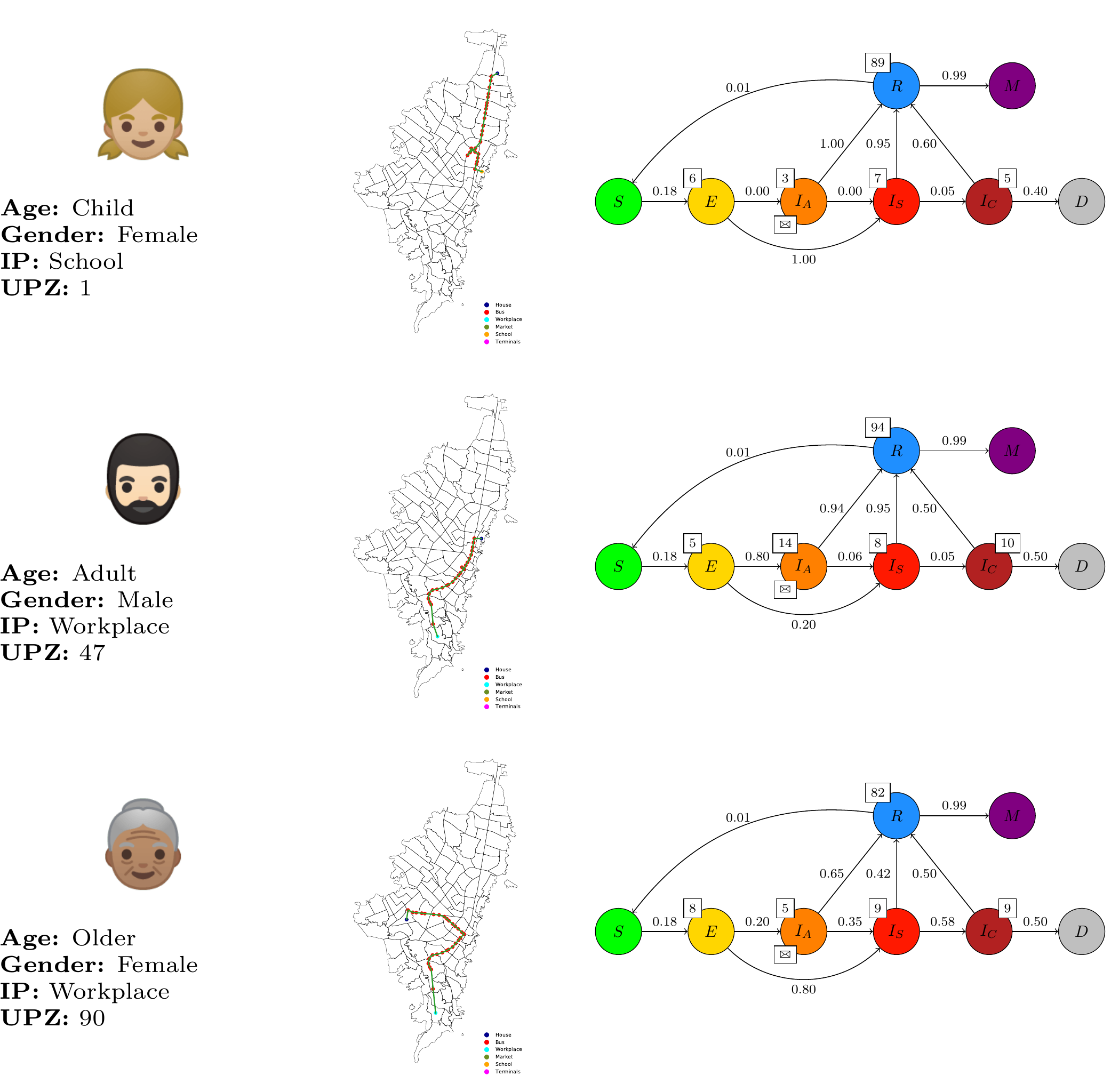

Supplement: S1 File — A repository containing the source code of the simulator and a technical report explaining the modeling methodology is available at INFEKTA github. (ZIP) [file pone.0245787.s001.zip › images/Fig3.tiff]

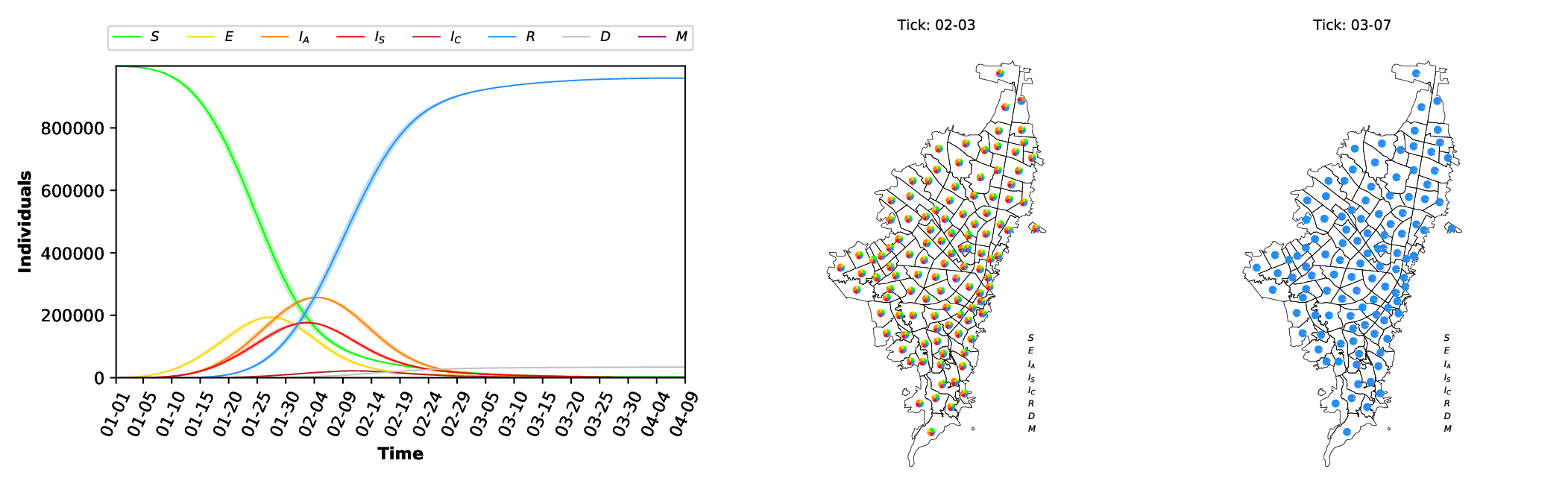

Supplement: S1 File — A repository containing the source code of the simulator and a technical report explaining the modeling methodology is available at INFEKTA github. (ZIP) [file pone.0245787.s001.zip › images/Fig4.tiff]

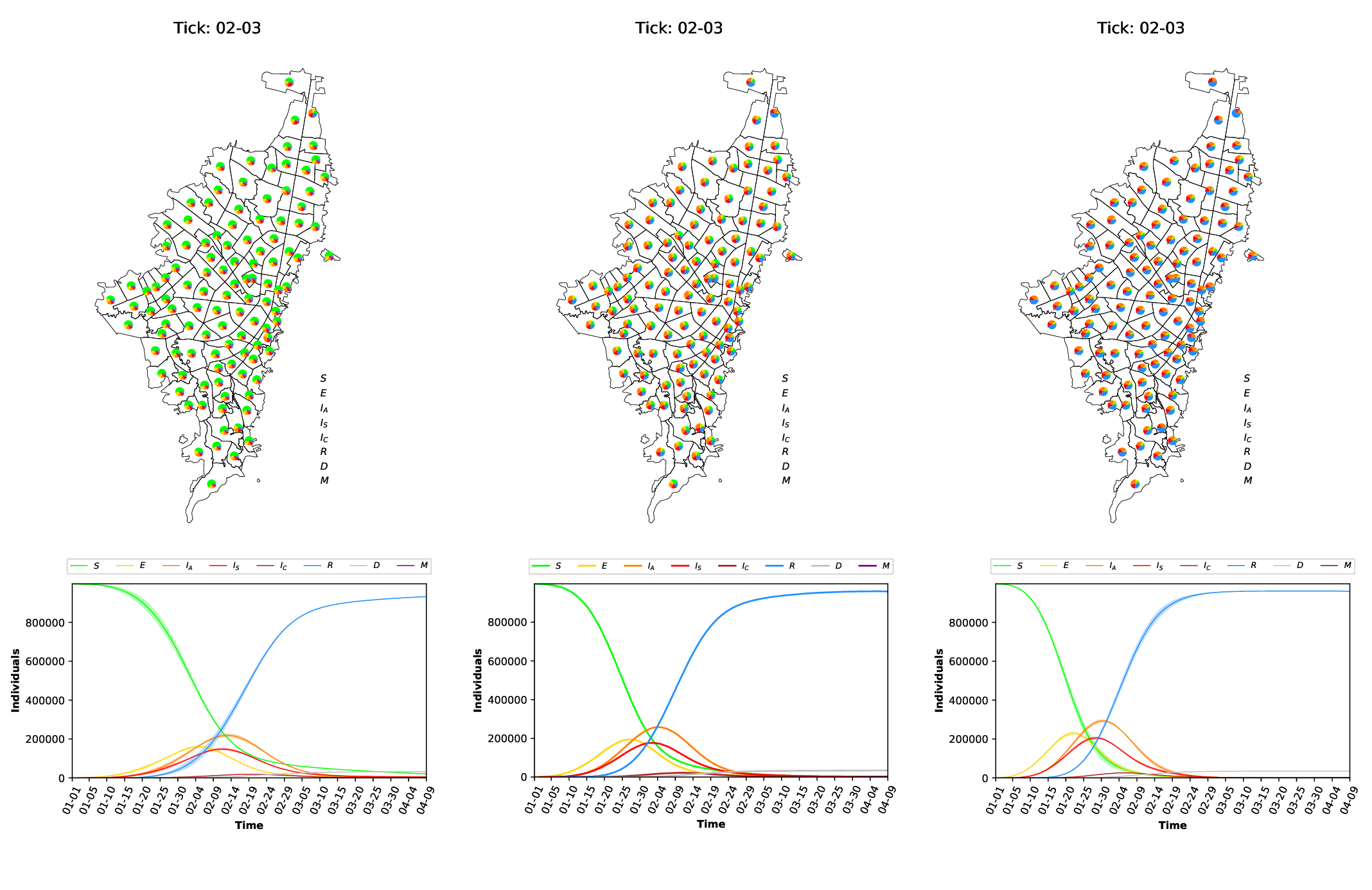

Supplement: S1 File — A repository containing the source code of the simulator and a technical report explaining the modeling methodology is available at INFEKTA github. (ZIP) [file pone.0245787.s001.zip › images/Fig5.tiff]

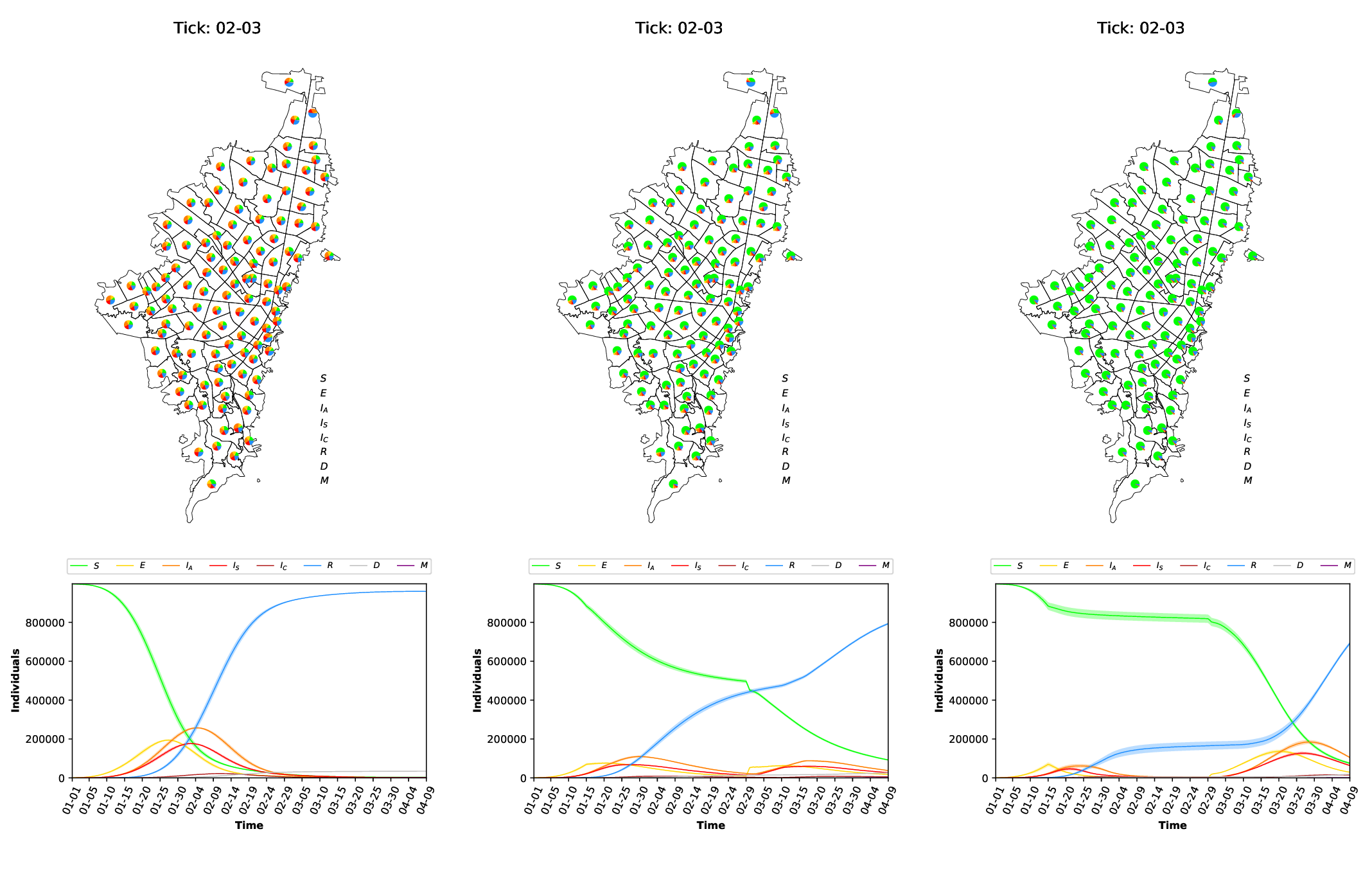

Supplement: S1 File — A repository containing the source code of the simulator and a technical report explaining the modeling methodology is available at INFEKTA github. (ZIP) [file pone.0245787.s001.zip › images/Fig6.tiff]

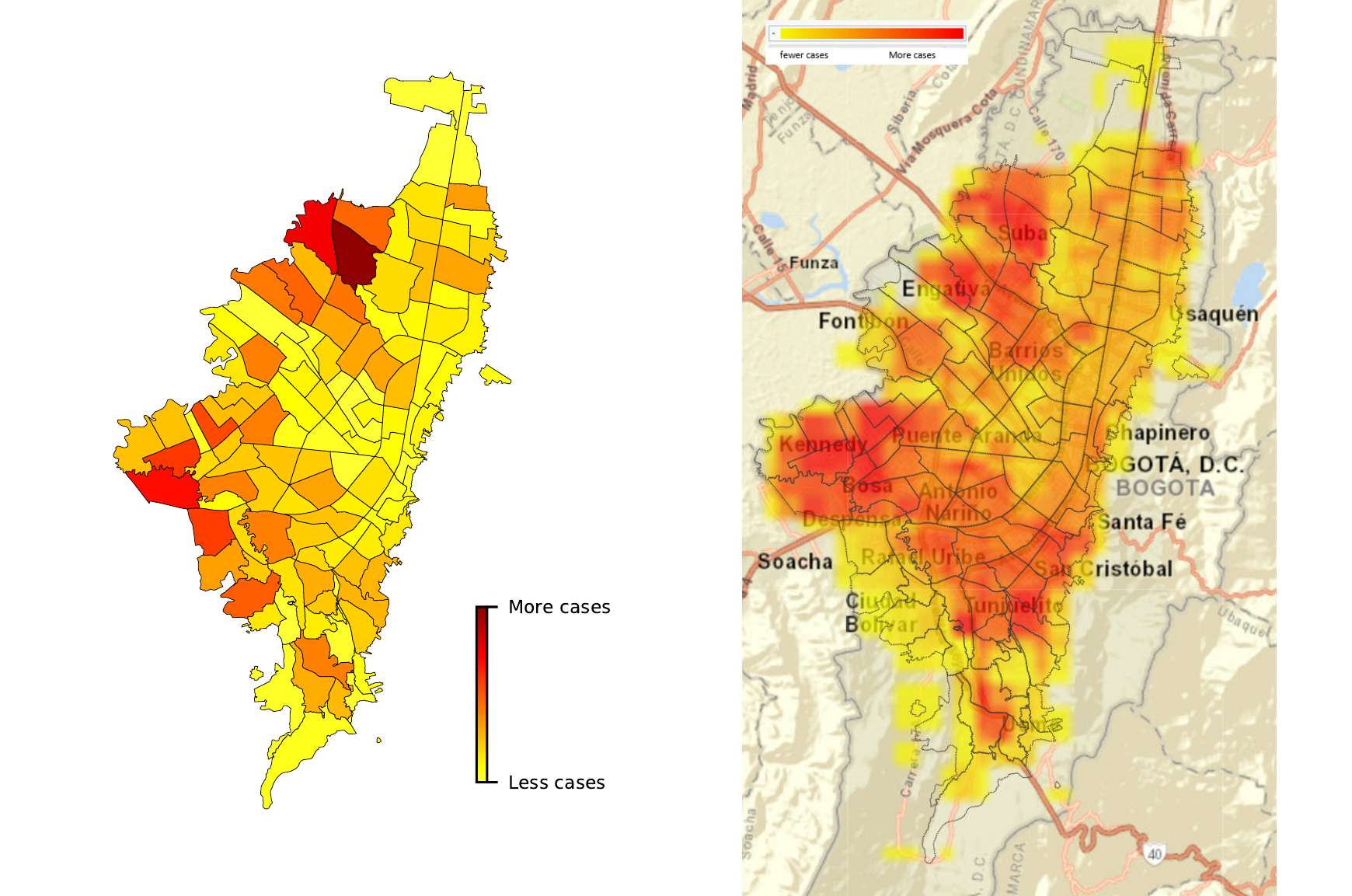

Supplement: S1 File — A repository containing the source code of the simulator and a technical report explaining the modeling methodology is available at INFEKTA github. (ZIP) [file pone.0245787.s001.zip › images/Fig7.tiff]

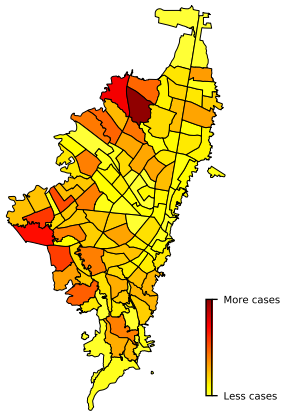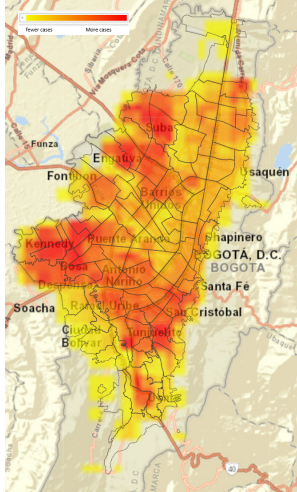

Supplement: S1 File — A repository containing the source code of the simulator and a technical report explaining the modeling methodology is available at INFEKTA github. (ZIP) [file pone.0245787.s001.zip › images/Fig7.pdf]
