# Supplementary material for: INFEKTA—An agent-based model for transmission of infectious diseases: The COVID-19 case in Bogotá, Colombia
Source: PLoS One. 2021 Feb 19;16(2):e0245787. doi: 10.1371/journal.pone.0245787 (PMC7894857; doi:10.1371/journal.pone.0245787)
Supplement: Attachment — Submitted filename: INFEKTA - Response to Reviewers.pdf [file pone.0245787.s002.pdf]

Dear Editor,

Many thanks for this opportunity to improve our contribution and paper description. Also many thanks to the reviewers for their constructive comments and suggestions. Please find below our responses to the reviewer's comments. Answers to the queries are in blue and literal changes added to the paper are in red.

Associate Editor:

1. Please ensure that your manuscript meets PLOS ONE's style requirements, including those for file naming. The PLOS ONE style templates can be found at https://journals.plos.org/plosone/s/file?id=wjVg/PLOSOne_formatting_sample_main_body.pdf and https://journals.plos.org/plosone/s/file?id=ba62/PLOSOne_formatting_sample_title_authors_affiliations.pdf

We review the template and style of PLOS ONE and consider that the proposed article meets the requirements of style.

2. Our internal editors have looked over your manuscript and determined that it is within the scope of our Cities as Complex Systems Call for Papers. This collection of papers is headed by a team of Guest Editors for PLOS ONE: Marta Gonzalez (University of California, Berkeley) and Diego Rybski (Potsdam Institute for Climate Impact Research). The Collection will encompass a diverse and interdisciplinary set of research articles applying the principles of complex systems and networks to problems in urban science. Additional information can be found on our announcement page: https://collections.plos.org/s/cities If you would like your manuscript to be considered for this collection, please let us know in your cover letter and we will ensure that your paper is treated as if you were responding to this call. If you would prefer to remove your manuscript from collection consideration, please specify this in the cover letter.

We think that the deadline has ended. However if it is possible to be added to this collection we will be glad if our paper is included there.

3. We note that Figures in your submission contain [map/satellite] images which may be copyrighted. All PLOS content is published under the Creative Commons Attribution License (CC BY 4.0), which means that the manuscript, images, and Supporting Information files will be freely available online, and any third party is permitted to access, download, copy, distribute, and use these materials in any way, even commercially, with proper attribution. For these reasons, we cannot publish previously copyrighted maps or satellite images created using proprietary data, such as Google software (Google Maps, Street View, and

Earth). For more information, see our copyright guidelines: http://journals.plos.org/plosone/s/licenses-and-copyright.

Figures were generated from scratch programmatically (using polygons, longitude and latitude), using open data referenced in the footnote of page 7 https://bogota-laburbano.opendatasoft.com/explore/dataset/poblacion-upz-bogota/table/. The Complex Network of Figure 2 that shows the associated complex network of connected places (nodes are places and edges are routes between places), the graph was drawn with Gephi.

Reviewer #1:

We thank the reviewer for appreciating our paper updates and for providing further constructive suggestions on how to improve the next version. We hope to have addressed these in a satisfactory manner, as explained below, point-by-point.

1. Size of the simulations and model capacity: The simulations presented in this study only include 1001 individuals. This is only a small fraction of the total population of Bogotá (8+ million). I'm concerned that this small size does not represent the density of the city. The synthetic population is geographically located according to the population density of the city, but with only 1001 individuals, the density of contacts is much lower than in reality. In this sense, the authors might be simulating a much less dense city than Bogotá. In addition, the small sample size would increase the impact of stochastic effects in the model, so that the epidemic could die out much sooner than expected.. This can explain why the authors found a similar effect of reducing social interactions by 75% than by 95%. I recommend looking into other agent-based models that don't simulate the entire population (https://www.mobs-lab.org/uploads/6/7/8/7/6787877/tracing_main_may4.pdf). Also, multiple simulations of the model could account for these stochastic effects, but the authors do not mention how many replicates were simulated with the same set of parameters.

We followed this recommendation and performed new experiments with 998.213 agents. Bogotá has a population of 7.412.566 and it is composed by 112 Zonal Planning Units (UPZ, for its acronym in spanish). Each UPZ belongs to one of the 19 urban districts in Bogotá. The number of places in each UPZ was generated based on the population density of each UPZ according to the data available in 2017 obtained from public information available by the District Planning Secretary: http://www.sdp.gov.co/gestion-estudios-estrategicos/estudios-macro/proyecciones-de-poblacion.

The following idea was added to clarify this query: The number of places in each UPZ is generated based on the population density of each UPZ according to the data available in 2017 [27]. Table 1 shows the amount of data generated for each type of place and for people, also the number of TM stations (Bus), and terminal transportation that we use in the simulation, and Table 2 shows a detailed information of the number of interest places generated by UPZ.

2. Epidemiological model

The transmission model might not be the appropriate for SARS-CoV-2. As I understand the model, exposed individuals are able to transmit the virus, which doesn't account for the incubation period of the virus. In addition to the incubation period, two important aspects of SARS-CoV-2 transmission should be included: asymptomatic transmission and pre-symptomatic transmission.

We have included a more detailed description of the model in section **Agents based model for infectious disease** and include a clock symbol that indicates a period of time able to change to another state. The reviewer is right. We include the Asymptomatic-Infected (IA) state after the exposed one to model the incubation period of the virus.

3. Place-specific contact rate/transmission probability

It's unclear to me if the transmission rates are the same for each place. If so, this might be unrealistic. The authors should account for place-specific contact rates to determine the transmission probability. For instance, if an individual works in a workplace with 1,000 employees, would INFEKTA consider that these contacts are as relevant for transmission as household contacts are?

We model fixed probabilities to model changes of state of an individual. Probabilities are applied if two individuals are close enough in the same place or transportation station. It means that more dense places implies more possible infected individuals.

In section Agents based model for infectious disease we added:

Each individual is modelled as an agent with an internal ”SEIR” state that represents its infectious disease state (severity and time in it) at any instant of time. Individuals interact between them, i.e., can infect or get infected, when they move at some instant to the same place, their SEIR states, and the infectious disease transmission rates, and how close they are (if they are in crowded places). Notice, the concept of crowded places is natural in INFEKTA and emerges from the agents interactions (eg. if more individuals move using the same transportation’s routes) and individual characteristics (e.g. children go to schools.).

4. Simulation experiments

The simulation experiments in this manuscript might not be enough to show the model capabilities. The current simulation experiments only take into consideration social distancing but the authors omit any specific public transportation intervention. Given that including public transportation routes is a strength of this work, the authors could perform some simulations that show the impact of changes in public transportation schedules and capacity. Current policies in Bogotá limit the capacity of transmilenio to about 35% of total capacity. The authors could explore similar strategies. Finally, it isn't clear how transmission occurs in the public transportation. Does it only happen in the terminals or bus routes?

Indeed, this aspect wasn't clear in the paper and we thank the reviewer for pointing it out. By now, we don't cover the capacity limit of transmilenio but model individual dynamics. As in the previous answer we add the following idea: Notice, the concept of crowded places is natural in INFEKTA and emerges from the agents interactions (eg. if more individuals move using the same transportation's routes) and individual characteristics (e.g. children go to schools.).

In the section individual setup we added the following:

Also, a sequence of activities was assigned randomly to each individual to define a diary routine. This was done according to the person's age and the hour of the day. For example, some agents Adolescence go to school, and some agents Adult go to work. Time to start routine -going from Home to IP and return- is randomly selected in the interval from 4h and 7h returning between the 17h and 20h.

Other comments:

- Abstract: "a Euclidean" should be "an Euclidean" (fixed, thank you)

- Abstract: What are the main results of this study?

The abstract was rewritten and main results of the study were included: This paper proposes an agent-based model, called INFEKTA, for simulating the transmission of infectious diseases, not only the COVID19, under social distancing policies.INFEKTA combines the transmission dynamic of an specific disease, (according to parameters found in the literature) with demographic information (population density, age, and genre of individuals) of geopolitical regions of the real town or city under study. Agents (virtual persons) can move, according to its mobility routines and the enforced social distancing policy, on a complex network of accessible places defined over an Euclidean space representing the town or city. The transmission dynamics of the COVID-19 under different

social distancing policies in Bogotá city, the capital of Colombia, is simulated using INFEKTA with one million virtual persons. A sensitivity analysis of the impact of those social distancing policies on the disease rates (Asymptomatic, Seriously, and Critically) indicates that it is possible to establish a not so 'hard' social distancing policy to achieve a significant reduction on the disease rate.

- Line 17-18. Try to avoid using "predicting", use estimating or predicting instead (fixed, thank you)

- Lines 24-25. Should "social separation" be "social distancing"? (fixed, thank you)

- Lines 26 - 77. This section might be unnecessarily long, and could be a part of the introduction. More importantly, I suggest that the authors elaborate more on the recent agent-based models available in the literature for COVID-19. It would be helpful to have more context on the current modeling approaches (Examples: Ferguson et al., or Vespigniani et al.) and how INFEKTA compares to those models.

Some other agent models were added in the following paragraph: In the past, ABMs have been employed to address various infectious diseases such as, a bioterrorist introduction of smallpox [9], control of tuberculosis [10], implementation of distancing measures and antiviral prophylaxis to control H5N1 influenza A (bird20flu) [11], design of vaccination strategies for influenza [12], devise evacuation strategies in the event of airborne contamination [13], and curtail transmission of measles through contact tracing and quarantine [14]. While this paper was under revision, some other works that include heterogeneous agents and social distancing were proposed to model COVID-19 [15, 16]. The proposed approach, called INFEKTA (Esperanto word for infectious), mainly differs from existing works in that it aims to generate individuals and a complex network of places based on the population density of a determined city.

- Lines 99-102. "No home is neighbor to any other home". Does this mean that neighborhood interactions among individuals only occur at public transportation stations?

The idea was extended as follows: Each individual has a Home and an IP. The closest distances are computed between each Home and IP and between these places with the closest PTS using their longitude and latitude. If the distance between a Home and IP is shorter than the distance to a PTS this Home will be connected directly to an IP instead of their closest PTS. Otherwise, the closest PTS is connected to each Home and IP respectively. Detailed information can be found in the Figure 2 of Section Virtual Space Setup.

- Line 145. Please explain Figure 1 with more detail.

Description of Figure 1, was extended and also the section to give more details to the model.

- Methods: It isn't clear to me if the parameters for transmission are fixed parameters or drawn from a distribution.

Parameters are fixed values extracted from a literature review.

We wrapped them in the transition rates and allowed modelers to change and play with different rates. Therefore, we set the initial values of these rates as shown in Table 4.

- Methods: It isn't clear to me which processes are random and which are deterministic in the model.

To address this comment, we added Table 4 that describes the initial parameters of Infekta based on a literature review. Probabilities and distribution of individuals are random and specific times are deterministic.

- Methods: Please explain in more detail how the synthetic population was created to match demographic characteristics of Bogotá.

We have clarified this aspect in the reply to comment #1.

- Lines 188-232: How do the authors account for the difference in the 1001 people in the simulation and the actual population of Bogotá? If the entire city is being simulated but only 1001 people are modeled, how does this affect the transmission dynamics? Is there any factor to account for the difference in synthetic and real density?

We have clarified this aspect in the reply to comment #1.

- Lines: 286-290. This could be caused by the small sample size. Stochastic effects can make a big difference with only 1001 people simulated.

We have clarified this aspect in the reply to comment #1. We thank the reviewer for this.

- Discussion: How does this model compare to other ABMs around the world?

Some other agent models were added in the following paragraph: In the past, ABMs have been employed to address various infectious diseases such as, a bioterrorist introduction of smallpox [9], control of tuberculosis [10], implementation of distancing measures and antiviral prophylaxis to control H5N1 influenza A (bird20flu) [11], design of vaccination strategies for influenza [12], devise evacuation strategies in the event of airborne contamination [13], and curtail transmission of measles through contact tracing and quarantine [14]. While this paper was under revision, some other works that include

heterogeneous agents and social distancing were proposed to model COVID-19 [15, 16]. The proposed approach, called INFEKTA (Esperanto word for infectious), mainly differs from existing works in that it aims to generate individuals and a complex network of places based on the population density of a determined city.

Reviewer #2: 1. In abstract, the sentence "In this paper, a general agent-based model, called…", I am not sure what authors mean by a general agent-based model. The word general sounds like a specific type of ABM. I suggest to explain what the word "general" means in this context, and if the ABM is a regular ABM, this word is unnecessary.

Word general has been removed from the abstract. And clarify this point

This paper proposes an agent-based model, called INFEKTA, for simulating the transmission of infectious diseases, not only the COVID19, under social distancing policies. INFEKTA combines the transmission dynamic of an specific disease, (according to parameters found in the literature) with demographic information (population density, age, and genre of individuals) of geopolitical regions of the real town or city under study. Agents (virtual persons) can move, according to its mobility routines and the enforced social distancing policy, on a complex network of accessible places defined over an Euclidean space representing the town or city.

2. I suggest that authors add at least one more line in the abstract where one central or maybe the most important result of the paper is presented. I believe that usually this attracts reader attention.

Thanks for this suggestion we rewrite the abstract as follows: This paper proposes an agent-based model, called INFEKTA, for simulating the transmission of infectious diseases, not only the COVID19, under social distancing policies.INFEKTA combines the transmission dynamic of an specific disease, (according to parameters found in the literature) with demographic information (population density, age, and genre of individuals) of geopolitical regions of the real town or city under study. Agents (virtual persons) can move, according to its mobility routines and the enforced social distancing policy, on a complex network of accessible places defined over an Euclidean space representing the town or city. The transmission dynamics of the COVID-19 under different social distancing policies in Bogotá city, the capital of Colombia, is simulated using INFEKTA with one million virtual persons. A sensitivity analysis of the impact of those social distancing policies on the disease rates (Asymptomatic, Seriously, and Critically) indicates that it is possible to establish a not so 'hard' social distancing policy to achieve a significant reduction on the disease rate.

3. In introduction, the sentence (line 10) "Complex systems are computational approaches that make use of computer-based algorithms to model dynamic interactions between

individuals agents…" is not accurate. Please, revise the complex systems concept. Actually, this sentence makes an inversion between complex systems and computer-based algorithms. Complex systems may be simulated by using computer algorithms, but the concept of such systems are wider than the one expressed in this sentence.

Indeed, the reviewer is right. We state the following: Many biological systems have been modeled in terms of complex systems since their collective behavior cannot be simply inferred from the understanding of their components.

Additionally, We replaced "Complex systems are computational approaches" for "Complex systems may be simulated by using computer-based algorithms to model dynamic interactions between individuals agents (e.g. persons, cells) or groups and their properties, within, and across levels of influence [6, 7]."

4. I think that the subsection "Complex Systems Approaches for Epidemic Models" (line 26) would make more sense if it is locate after the subsection "Compartmental Model" (line 37). Thus, the new sequence would be "Compartmental Model" and after this subsection, "Complex Systems Approaches for Epidemic Models". My argument is that initially authors should explain what compartmental models are and the limitations they might have. After that, an introduction about complex systems approach an then, the ABM subsection.

Since for our model the most important part is the complex network of places more than the compartmental model, we decide to adopt the current structure. We added examples and Figures like Figure 3 to clarify why we adopt this subsection structure.

5. First paragraph of the subsection "Agent-based models for infectious disease" is fine, although I think authors could add a comment about that agents in an ABM model may take decisions based on some system conditions or rules.

In section Agents based model for infectious disease we added:

Each individual is modelled as an agent with an internal "SEIR" state that represents its infectious disease state (severity and time in it) at any instant of time. Individuals interact between them, i.e., can infect or get infected, when they move at some instant to the same place, their SEIR states, and the infectious disease transmission rates, and how close they are (if they are in crowded places). Notice, the concept of crowded places is natural in INFEKTA and emerges from the agents interactions (eg. if more individuals move using the same transportation's routes) and individual characteristics (e.g. children go to schools.).

6. Lines 67 to 70 are repeating what was already mentioned about ABMs and complex system, then I suggest, as these lines a redundant, they could be deleted. (fixed, thank you)

7. The sentence in lines 73 to 75 are not very clear to me. Is this what authors meant: In this way, each virtual individual includes its health state, as implemented in a SEIR model, which is fundamental for the infectious disease transmission?

We added the paragraph presented as the answer to comment 5 to clarify the idea.

8. In Section “INFEKTA Agent-Based Model”, for the sake of simplicity, the first paragraph should be reduced to “Our agent-based model of infectious disease propagation, called INFEKTA, consists of five-layer components:”. The remaining part of line 80 until line 85 may be delete because each one of the model layers are explained right after the colon. Therefore, I think that the text would benefit of clarity if each layer does not have subsection, maybe items or subitens. For instance, the layer “Space” should explain “Place (Node)” and “Neighbor (Edge)” as part of the same text the item “Space”. The same idea may be applied to explanation of the other remaining layers. (fixed, thank you)

9. Lines 87 to 90: important concepts of the model are presented in these lines. Part of them are between parentheses, so I suggest that they be place between commas, because they are fundamental to understand the concept that is being exposed. (fixed, thank you)

10. Neighbor (Edge), line 98: concept is simple but the relations (neighborhood) among them are not very clear in the explanation. Authors may consider two options: re-write the sentence or add a simple figure representing each concept and the relations among them.

The sentence was clarified as follows: Each individual has a Home and an IP. The closest distances are computed between each Home and IP and between these places with the closest PTS using their longitude and latitude. If the distance between a Home and IP is shorter than the distance to a PTS this Home will be connected directly to an IP instead of their closest PTS. Otherwise, the closest PTS is connected to each Home and IP respectively. Detailed information can be found in the Figure 2 of Section Virtual Space Setup.

11. Line 100, the footnote: replace "in" by "In". (fixed, thank you)

12. Line 119. What is “Going hour”? There is no definition for it in the text.

Definition was added: Going hour is the time an individual moves from Home to a IP. In section individuals setup the following was added: Time to start routine -going from Home to IP and return- is randomly selected in the interval from 4h and 7h returning between the 17h and 20h.

13. Line 135. In the case of covid-19, as literally everybody is susceptible to the SARS-Cov-2 virus, the health state “Immune (M)” only makes sense when the individual is recovered from the disease. However, the “Recovered (R)” health state is also defined in the model. Then, my question is: what is the difference between the two health states? Besides, in line

142, authors say “INFEKTA introduces both the M state since some individuals are naturally immune”. In the literature, one can find that, if so, only a few persons have some partial immunity against SARS-Cov (not SARS-Cov-2), however, this is not a lifetime imunnity and this outbreak happened in the early 2000’s. Moreover, the amount of people infected with SARS-Cov is so small and geographically restricted that does not justify to model a pandemic caused by SARS-Cov-2 considering that some individuals have any prior immunity. Then, this part of the text has to be re-written.

Figure 1 shows clearly the immunity is acquired only after the individual has recovered from the infection: this is the correct dynamics of covid-19 outbreak and, as recovered individuals have acquired immunity immune, a new compartment called “M” is not necessary.

In addition, in Fig.1, there is a line from R compartment to S compartment, meaning that recovered individuals become susceptible again. So far, in the literature about covid-19 pandemic there is no evidence that recovered individuals become susceptible again. Thus, this fact implies that this model has to be considered incorrect and for this reason, until this correction is made, I suggest the paper is rejected.

In the abstract we added the following: This paper proposes an agent-based model, called INFEKTA, for simulating the transmission of infectious diseases, not only the COVID19, under social distancing policies. When presenting Figure 1 we clarified this aspect mentioned by the reviewer: Figure 1 shows the general transition dynamics of any infectious disease at the individual level in INFEKTA. This model can be adjusted to any specific infectious disease by setting some of the probabilities to specific values. For example, if there is no evidence that recovered individuals become immune or susceptible again, such probabilities can be set to 0.0.

---

6. PLOS authors have the option to publish the peer review history of their article (what does this mean?). If published, this will include your full peer review and any attached files.

If you choose “no”, your identity will remain anonymous but your review may be made public.

Do you want your identity to be public for this peer review? For information about this choice, including consent withdrawal, please see our Privacy Policy.

Reviewer #1: Yes: Guido Camargo España

Reviewer #2: No

[NOTE: If reviewer comments were submitted as an attachment file, they will be attached to this email and accessible via the submission site. Please log into your account, locate the manuscript record, and check for the action link "View Attachments". If this link does not appear, there are no attachment files.]

While revising your submission, please upload your figure files to the Preflight Analysis and Conversion Engine (PACE) digital diagnostic tool, https://pacev2.apexcovantage.com/. PACE helps ensure that figures meet PLOS requirements. To use PACE, you must first register as a user. Registration is free. Then, login and navigate to the UPLOAD tab, where you will find detailed instructions on how to use the tool. If you encounter any issues or have any questions when using PACE, please email PLOS at figures@plos.org. Please note that Supporting Information files do not need this step.

.
