# Supplementary material for: INFEKTA—An agent-based model for transmission of infectious diseases: The COVID-19 case in Bogotá, Colombia
Source: PLoS One. 2021 Feb 19;16(2):e0245787. doi: 10.1371/journal.pone.0245787 (PMC7894857; doi:10.1371/journal.pone.0245787)
Supplement: Attachment — Submitted filename: INFEKTA - Response to Reviewers (V.2.0).pdf [file pone.0245787.s003.pdf]

Dear Editor,

First, we would like to thank you for providing further constructive suggestions on how to improve the next version. We provide a few answers and clarifications for each outstanding comment below. Answers to the queries are in blue and literal changes added to the paper are in red.

Reviewer #1:

Many thanks for providing further constructive suggestions on how to improve the next version. We hope to have addressed these in a satisfactory manner, as explained below, point-by-point.

1. There is still an issue with my first comment regarding the stochasticity nature of the model and the population size. In terms of the stochasticity, the authors only report the mean of their results, do not include any uncertainty. For the population size, I understand the need for simplification in the population size, but the authors should at least add in the discussion what impact does this underestimation have in their results. For instance, transmilenio buses or stations would be less crowded than expected, given that there are fewer people in the model. In general, the authors should add a section in the discussion listing the model limitations.

Standard deviation data were added to Figures 4, 5 and 6 that corresponds to the experiments performed.

Also, we add a paragraph with the limitations in lines 250-256:

Despite the usefulness of the INFEKTA, there are some limitations. Since there are fewer people in the model (respect to real people in Bogotá), \textit{Transmilenio} stations would be less crowded than expected and the model could underestimate the transmission of the disease. Further, the model does not cover individual walking from homes to PTS because contact points correspond to a complex network presented in Figure 2. Finally, the routines that each individual has in the simulation are equal day-to-day (i.e., Monday to Sunday with the same routine), this could influence the stochasticity nature of the model.

2. The authors added a couple of other models in their introduction, which I appreciate. I don't agree that this model differentiates in having a complex network of places and population density. Several of the agent-based models that have been available for a long time to simulate COVID-19 include realistic representations of human activities and their population demographic characteristics. Including public transportation might be a more unique characteristic of this model. Also, there are

many more than two abm for COVID-19, and have been released before this manuscript was under review.

Thank you for this comment. Lines 23 - 28 were updated:

In our literature review, some other novel works that include heterogeneous agents and social distancing were proposed to model COVID-19 [15],[16]. The proposed approach, called INFEKTA (Esperanto word for infectious), mainly differs from existing works in that it aims to generate individuals and a complex network of places based on the population density of a determined city including individual interaction in public transportation means.

3. For future reviews. Please add the line numbers where the changes have been made. It is difficult to follow the responses without the lines where the changes were made.

(Thanks for your advice)

4. Abstract: `not so hard` -> please use specific and descriptive words.

At the end of the Abstract we change the word 'hard' by 'medium' and give the corresponding definition (The full description of the 'medium' social-distance policy is in the line 251, section Social Distancing Rule Setting): A sensitivity analysis of the impact of social distancing policies indicates that it is possible to establish a 'medium' (i.e., close 40\% of the places) social distancing policy to achieve a significant reduction in the disease transmission.

5. Line 306-309. the statement "indicate that INFEKTA may be a valuable asset for researchers and public health decision-makers for projecting future scenarios when applying social distancing policy rules" is not supported by their results. 1. The authors do not contrast their simulations to any data in the manuscript to calibrate model parameters, 2. The authors do not make any projections and contrast them with epidemiological data in Bogotá. If the model is not validated with data, how can it be used to project future scenarios? I think the model could be used to explore scenarios, but not to project or predict.

Word projecting was changed by exploring (lines 325-328) and figure 7 was added as shown in the paper (lines 297-306):

Although our intention was not to predict geographic spread for the city, we observed similarities between the total Seriously-Infected cases in INFEKTA (we assume that individuals in Seriously-Infected state are cases tested in Bogotá) and the current concentration of COVID-19 cases confirmed in Bogotá, see Figure \ref{fig:mapCOVID}. The results show how UPZ with more cases found with INFEKTA matches with geographic

areas with more COVID-19 cases. Then, how population distribution is generated from real density data of Bogotá, INFEKTA can be useful to explore policies by Zonal Planning Units (UPZ) or territorial divisions of a selected place providing to recommend actions for before, during, and after pandemic i.e., in planning and coordination efforts through leadership and coordination across sectors.
